# Orm2 promotes nitrogen-induced sphingolipid production and endocytosis via Orm1 phosphorylation

**DOI:** 10.1016/j.jlr.2025.100924

**Published:** 2025-10-08

**Authors:** Jihui Ren, Yusuf A. Hannun

**Affiliations:** 1 Department of Medicine, Stony Brook University, Stony Brook, NY, USA; 2 Stony Brook Cancer Center, Stony Brook University, Stony Brook, NY, USA; 3 Northport Veterans Affairs Medical Center, Northport, NY, USA

**Keywords:** endocytosis, nitrogen-sensing, Orm2, sphingolipids, Ypk1 signaling

## Abstract

The Orm family of proteins inhibit serine palmitoyltransferase—the enzyme that catalyzes the first step in sphingolipid synthesis. In *S. cerevisiae*, the two Orm proteins are thought to function redundantly in suppressing sphingolipid production. Here, we show that Orm2, in contrast, *promotes* production of complex sphingolipids by regulating Ypk1-dependent phosphorylation of Orm1 through control of long-chain base (LCB) levels, the initial precursors in the sphingolipid biosynthesis pathway. Using targeted lipidomic analysis of *orm1*Δ versus *orm2*Δ strains, the results showed that Orm1 regulates complex sphingolipid levels, whereas Orm2 primarily modulates the production of LCBs. We then show that reduced Orm1 phosphorylation in *orm2*Δ cells was mediated by LCB-dependent inactivation of AGC family protein kinase Ypk1. We further demonstrate that this Orm2–LCBs–Ypk1–Orm1 regulatory module is responsive to nitrogen availability, promoting sphingolipid synthesis under nitrogen-rich conditions. Functionally, this pathway is required for nitrogen-induced endocytosis of the general amino acid permease Gap1. Together, our findings reveal that Orm2 governs sphingolipid production and downstream endocytic events via a nitrogen-responsive LCBs-Ypk1-Orm1 signaling pathway, linking nitrogen status to sphingolipid metabolism and membrane trafficking.

Sphingolipids (SPLs) are major components of the plasma membrane, where they play critical structural and functional roles (1). Intermediate products of SPL metabolism-including long chain bases (LCBs), ceramides and LCB phosphates (LCB-1-P)- also serve as signaling molecules with distinct cellular functions. SPL biosynthesis is a multi-step process that is conserved across species (2). The first enzyme in the de novo pathway is serine palmitoyltransferase (SPT), which catalyzes the production of LCBs. LCBs are subsequently converted into ceramides by ceramide synthases (CerSs). Ceramides then translocate from the endoplasmic reticulum to the Golgi apparatus, where they are converted into complex SPLs and transported to the plasma membrane. In this study, the term 'SPLs' refers specifically to complex sphingolipids.

The Orm family proteins are integral components of the SPT complex (3, 4, 5, 6, 7). By binding to the catalytic subunits, Orm proteins inhibit SPT activity. In the yeast *S. cerevisiae*, the binding and inhibitory effects of Orm proteins on SPT are regulated by their phosphorylation status, which is mediated by the AGC family protein kinases, Ypk1 and Ypk2, which directly phosphorylate the Orm proteins, weakening their interaction with the catalytic subunits and thereby relieving SPT inhibition (8, 9, 10, 11). In this study, we refer to the phosphorylated form of Orm proteins as *inactivated*, as phosphorylation impairs their ability to inhibit SPT. Conversely, the unphosphorylated form is referred to as *activated*, as it enhances the capacity to inhibit SPT. Although LCBs, the initial SPL precursors, are the direct products of SPT activity, Orm proteins are generally thought to regulate complex SPL production under the assumption that LCBs efficiently flow through the de novo synthesis pathway and are converted into complex SPLs. Yeast Orm proteins have traditionally been viewed as regulators of SPL homeostasis based on the observation that their phosphorylation increases following treatment with SPT inhibitor, myriocin—presumably to relieve inhibition and restore SPT activity in response to SPL depletion (3). The Torc2–Ypk1 signaling pathway has been proposed to mediate this feedback response by sensing changes in complex SPL levels at the plasma membrane and modulating phosphorylation of both Orm proteins to compensate for SPL gain or loss (3, 9, 12, 13, 14).

Many organisms, including yeast *S. cerevisiae* and humans, possess multiple Orm family proteins- Orm1 and Orm2 in yeast, and ORMDL1, ORMDL2 and ORMDL3 in humans. It is generally believed that these Orm family members have overlapping functions in negatively controlling SPL production. This conclusion is primarily based on the observations that deletion of a single *ORM* or *ORMDL* gene has only a modest effect of SPL levels, whereas deletion of all *ORM* or *ORMDL* genes leads to a dramatic accumulation of SPLs or SPL intermediates (4, 15, 16). However, emerging evidence suggests that homologous Orm proteins within a species may also perform distinct functions. In yeast, specific phenotypes such as hypersensitivity to oleic acid and tunicamycin are observed in *orm2*Δ but not *orm1*Δ mutants (4, 17). Additionally, overexpression of Orm2, but not Orm1, confers resistance to ion toxicity (18) and *ORM2*, but not *ORM1*, is upregulated in response to ER stress induced by tunicamycin or DTT (4). Moreover, only Orm2 is subject to regulation via ubiquitin-mediated endosome and Golgi-associated degradation (EGAD) (10). In humans, among the three *ORMDL* genes, only *ORMDL3* has been identified as an asthma susceptibility gene (19, 20), and it is also associated with Crohn’s disease (21, 22), type I diabetes (23), and obesity (24). However, no significant differences have been observed among the three ORMDL proteins in their ability to regulate SPL production. Phylogenetic analysis based on multiple sequence alignments of Orm family proteins reveals that ORMDL1, ORMDL2 and ORMDL3 form distinct clades, each grouping with their respective orthologs in human, mouse and zebrafish (Fig. 1). In contrast, species with fewer Orm proteins—such as Saccharomyces cerevisiae and Arabidopsis thaliana (two members), or Drosophila melanogaster and Schizosaccharomyces pombe (one member)—exhibit greater genetic divergence. This sequence analysis suggests that functional diversification among Orm family proteins may be an ancestral trait.

**Fig. 1:**
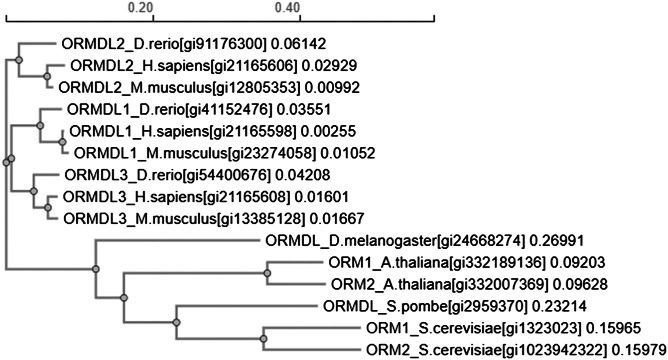
Phylogenetic tree of ORM-like proteins from various organisms. Protein sequences of ORM-like homologs from the indicated species were aligned using the Clustal Omega multiple sequence alignment tool (EMBL-EBI) to construct the phylogenetic tree. For each sequence, the GenInfo Identifier (gi) number follows the species name. The number at the end of each branch tip indicates the branch length with the scale bar on top.

In a recent study, we investigated the effects of Orm1 and Orm2 on sphingolipid metabolism and flux in *S. cerevisiae* using *orm1/2*Δ yeast mutant, in which both *ORM1* and *ORM2* were deleted (25). We found that loss of both *ORMs* had a greater impact on LCB levels than on complex SPLs. Our data also showed that Orm proteins regulate Ypk1 signaling and downstream endocytic events by modulating LCB levels. These findings suggested that Orm proteins have functions beyond maintaining complex sphingolipid homeostasis and raise the question of how Orm1 and Orm2 regulate Ypk1 signaling—whether they act redundantly or perform distinct roles in this process.

In this study, we set out to investigate the functional differences between the yeast Orm1 and Orm2 proteins. We first found that the SPLs were differentially affected by deletion of *ORM1* (*orm1*Δ) versus *ORM2* (*orm2*Δ). As expected, *orm1*Δ cells showed increased SPL production. In contrast, *orm2*Δ cells accumulated LCBs without an increase in complex SPL levels due to a defect in their conversion into ceramides. We then observed that Ypk1-dependent Orm1 phosphorylation was reduced in *orm2*Δ cells in an LCB-dependent manner. To assess the physiological relevance of this Orm2–LCB–Ypk1-Orm1 regulatory module, where Orm2 is required for Orm1 phosphorylation and subsequent SPT activation, we examined its role in a central nutrient response program: yeast’s adaptation to nitrogen availability. We found that SPL production increased upon nitrogen repletion and that this increase was required for efficient endocytosis of the general amino acid permease, Gap1. During the transition from nitrogen-poor to nitrogen-rich conditions, Orm2 was dephosphorylated (activated) and was required for subsequent Orm1 phosphorylation (inactivation). Importantly, this Orm2-dependent inactivation of Orm1 was necessary for both nitrogen-induced SPL synthesis and Gap1 endocytosis. Taken together, our results demonstrate that Orm2 promotes SPL production by facilitating Orm1 inactivation through an LCB-Ypk1 signaling pathway in response to nitrogen abundance.

## Materials and Methods

### Yeast strains and growth conditions

All *Saccharomyces cerevisiae* strains used in this study are listed in Table 1. Yeast were cultured at 30°C in either liquid YPD (yeast extract, peptone, and dextrose) or synthetic defined (SD) medium, composed of 0.67% yeast nitrogen base without amino acids or carbohydrates, 2% glucose, and supplemented with the following amino acids/pyrimidine: 120 mg/L leucine, 20 mg/L uracil, 20 mg/L histidine, and 20 mg/L methionine. Nitrogen-poor medium (–N) consisted of 0.17% yeast nitrogen base without amino acids, ammonium sulfate, or carbohydrates (USBiological), 2% glucose, and the necessary amino acids to support the growth of auxotrophic strains. Nitrogen-rich medium (+N) was prepared by supplementing –N medium with 50 mM ammonium sulfate. Gene deletions were performed using homologous recombination to replace target genes with antibiotic resistance or auxotrophic selection markers, as previously described (26). Genomic modifications—such as point mutations or protein tagging—were introduced using a CRISPR-Cas9 system. A haploid strain with the gene of interest replaced by KanMX was co-transformed with a plasmid expressing Cas9 and a guide RNA targeting KanMX (pRS425-Cas9-KanMX), along with a PCR product containing the desired gene modification. Surviving colonies were screened by sequencing to confirm correct integration.

**Table 1:** Yeast strains used in this study

Strain	Genotype	Source/reference
BY4741	MATa his3Δ1 leu2Δ0 met15Δ0 ura3Δ0	Dharmacon™
*orm1*Δ	BY4741 *orm1*Δ::KanMX	Dharmacon™
*orm2*Δ	BY4741 *orm2*Δ::URA3	This study
*Flag-Orm1*	BY4741 3XFlag-Orm1	Reference (3)
*orm2*Δ *Flag-Orm1*	BY4741 3XFlag-Orm1 *orm2*Δ::URA3	This study
*orm2*Δ*tsc3*Δ *Flag-Orm1*	BY4741 3XFlag-Orm1 *orm2*Δ::URA3 *tsc3*Δ::KanMx	This study
*orm2*Δ*lcb3*Δ *Flag-Orm1*	BY4741 3XFlag-Orm1 orm2Δ::URA3 lcb3Δ::KanMx	This study
*lac1*Δ *Flag-Orm1*	BY4741 3XFlag-Orm1 lac1Δ::KanMx	This study
*cka2*Δ *Flag-Orm1*	BY4741 3XFlag-Orm1 cka2Δ::KanMx	This study
*elo3*Δ *Flag-Orm1*	BY4741 3XFlag-Orm1 elo3Δ::KanMx	This study
*Ypk1*Δ *Flag-Orm1*	BY4741 3XFlag-Orm1 ypk1Δ::KanMx	This study
*Flag-Orm2*	BY4741 3XFlag-Orm2	This study
GAP1-GFP	BY4741 GAP1-GFP::His3MX6	Dharmacon™
*Ypk1*ΔGAP1-GFP	GAP1-GFP *ypk1*Δ::KanMX	This study
*orm1*^*AAA*^GAP1-GFP	GAP1-GFP *orm1*^*S51AS52AS53A*^	This study
*orm2*^*AAA*^GAP1-GFP	GAP1-GFP *orm2*^*S46AS47AS48A*^	This study

### Lipid analysis of LCBs and ceramides

Lipid extraction and quantification of long-chain bases (LCBs) and ceramides by HPLC-ESI-MS/MS were performed as previously described, with minor modifications (27, 28). Briefly, yeast cells cultured in SD, nitrogen-poor, or nitrogen-rich medium were harvested by centrifugation. Internal standards—C17-sphingosine (for LCBs) and C18:1 ceramide (for ceramides), each at 50 pmol—were added to the cell pellets (Avanti® Polar Lipids, Inc.). Lipids were extracted, dried, and resuspended in 200 μl of mobile phase B (0.2% formic acid and 1 mM ammonium formate in methanol). A 10 μl aliquot of each sample was injected into an HPLC system coupled to an electrospray ionization (ESI) mass spectrometer operating in positive multiple reaction monitoring (MRM) mode. The following MRM transitions were used.

• C17-sphingosine (286.2/268.2)
• C18:1 ceramide (564.5/264.3)
• 3-ketodihydrosphingosine (3KDS, 300.2/270.3)
• Dihydrosphingosine (DHS, 302.3/60.1)
• Phytosphingosine (PHS, 318.3/300.3)
• Phytosphingosine-1-phosphate (PHS-1-P, 398.2/300.3)
• C26-phytoceramide (C26-PHC, 696.4/282.3)
• C26-hydroxyphytoceramide (C26-PHC-OH, 712.7/282.2)

Data were acquired and analyzed using Xcalibur software. The relative abundance of each sphingolipid species was calculated by normalizing the peak area of each analyte to that of the internal standard and to the number of cells processed. All detected peak areas fell within the linear range of their respective standard curves.

### Lipid analysis of complex SPLs

Quantification of complex SPLs by HPLC-ESI-MS/MS was performed as previously described (25). Briefly, approximately 0.2–2 × 10^8^ yeast cells were harvested and subjected to lipid extraction as described earlier (28). C26-ceramide-IPC (44:1:2) was added to each sample at 50 pmol as an internal standard. Dried lipid extracts were resuspended in 150 μl of 1 mM ammonium acetate in 99% methanol. A 10 μl aliquot was injected into an HPLC system coupled to an electrospray ionization (ESI) mass spectrometer operating in negative multiple reaction monitoring (MRM) mode. The following MRM transitions were used for detection.

• C26-Cer-IPC (918.5/240.9)
• C26-PHC-OH-IPC (952.6/240.9)
• C26-PHC-OH-MIPC (1114.5/421.1)
• C26-PHC-OH-M(IP)_2_C (677.9/240.9)

Data were acquired and processed using Xcalibur software. Quantification was performed by normalizing the peak areas of detected sphingolipids to both the internal standard and the number of cells analyzed.

### Measurement of deuterated serine flux into the SPL pathway

Measurement of incorporation of D2-Ser into SPLs was performed as previously described (25). Briefly, 7.6 mM L-serine (3,3)-D_2_ (Cambridge Isotope Laboratories, Inc.) was added to the yeast culture. Approximately 10 ml of cells were collected at 0, 15, 30, 60, 90, and 120 min post-labeling. Lipid extraction and HPLC-ESI-MS/MS analysis were performed. The following mass transition parameters were used for D_2_-labeled sphingolipids.

• D_2_-3KDS (302.2/270.3)
• D_2_-DHS (304.3/62.1)
• D_2_-PHS (320.3/302.3)
• D_2_-PHS-1-P (400.2/302.3)
• D_2_-C26-PHC (698.2/680.2)
• D_2_-C26-PHC-OH (714.7/284.2)

The relative abundance of each D_2_-labeled sphingolipid species was calculated as described in the “LCB and ceramide lipid analysis” section, with the modification that background signals from corresponding endogenous compounds containing two C^13^ isotopes were subtracted from the detected signal.

### Western blot

Whole-cell yeast extracts were resolved by SDS-PAGE (10% acrylamide gels), with or without the addition of 40 μM Phos-Tag™ (FUJIFILM Wako Chemicals) and 80 μM MnCl_2_. Gels were run at 100 V and transferred to nitrocellulose membranes. Membranes were probed with the following primary antibodies.

• Mouse monoclonal anti-FLAG M2 (1:5000, Cat# F3165, MilliporeSigma)
• Rat monoclonal anti-α-Tubulin (YOL 1/34) (1:2500, Cat# NB100-1639, Novus Biologicals)
• Rabbit monoclonal anti-phospho-PKC (pan)ζ T410 (used for detecting phospho-Ypk1 at T504) (1:1000, Cat# 20605, Cell Signaling Technology)
• Rabbit polyclonal anti-Ypk1 (1:1000), kindly provided by Dr Mitsuaki Tabuchi (29).
• Rabbit monoclonal anti-GFP (1:1000, Cat# 2956, Cell Signaling Technology)

Following incubation with primary antibodies, membranes were washed and incubated with species-specific horseradish peroxidase (HRP)-conjugated secondary antibodies (anti-mouse, anti-rabbit, or anti-rat IgG; 1:5000, Jackson ImmunoResearch). Protein bands were visualized using Pierce™ ECL Western Blotting Substrate (Thermo Fisher) and imaged using the ChemiDoc Imaging System (Bio-Rad).

### Endocytosis assay of Gap1-GFP

Yeast cells expressing Gap1-GFP were cultured in nitrogen-free (–N) medium for 24 h prior to induction with 50 mM ammonium sulfate. Cells were harvested at 0, 15, 30, 60, 90, and 120 min following ammonium addition. At each time point, cells were mounted on glass slides and immediately imaged using an Olympus BX53 fluorescence microscope (Olympus Corporation of the Americas), equipped with a ∼488 nm excitation filter and a 500–550 nm emission filter.

## Results

### Distinct effects of *orm1*Δ and *orm2*Δ on SPL metabolism

**Fig. 2:**
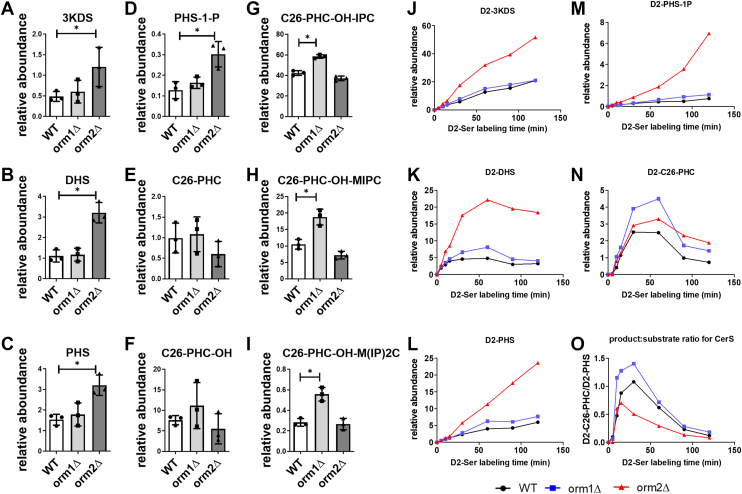
Deletions of *ORM1* and *ORM2* differentially affect SPLs. A-I: Distinct SPL profiles in *orm1Δ* versus *orm2Δ* yeast. Whole-cell lipids were extracted and analyzed by HPLC-ESI-MS/MS. The relative abundance of LCBs (A, B, C), LCB-1-P (D), major ceramides (E, F), and complex SPLs (G, H, I) was quantified based on peak area of the mass spec chromatogram of the indicated compounds, normalized to internal standard and cell number. Data represent the mean ± SD from three biological replicates. ∗*P* < 0.05 by Student’s *t* test. (J-O) *orm2Δ* is defective in converting LCBs into ceramides. Incorporation of deuterated L-Ser (3,3)-D2 (D2-Ser, 7.6 mM) into labeled SPL species was measured in WT (black circles), *orm1Δ* (blue squares), and *orm2Δ* (red triangles) strains. This is a representative of three independent repeats. Time-course analysis of D2-labled LCBs (J, K, L), LCB-1-P (M), and ceramides (N) was performed using HPLC-ESI-MS/MS. O: The ratio of D2-C26-PHC and D2-PHS at each time point was used to estimate CerS activity. Abbreviations: 3KDS: 3-ketodihydrosphingosine; DHS: dihydrosphingosine; PHS: phytosphingosine; PHS-1-P: phytosphingosine-1-phosphate; C26-PHC: C26-phytoceramide; C26-PHC-OH: hydroxy C26-phytoceramide; C26-PHC-OH-IPC: inositol phosphoryl C26-PHC-OH; C26-PHC-OH-MIPC: mannose-inositol-phosphoryl C26-PHC-OH; M(IP)2C: mannose-(inositol-P)2-C26-PHC-OH.

It is generally believed that Orm proteins regulate sphingolipid (SPL) production by controlling long-chain base (LCB) synthesis, under the assumption that LCBs flow freely through the de novo pathway and are then converted into complex SPLs. To directly test the effects of Orm proteins on SPL production, we comprehensively examined SPL levels in wild-type (WT), *orm1*Δ, and *orm2*Δ yeast strains. Using targeted HPLC-ESI-MS/MS methods, we quantified the major yeast SPL intermediates, including LCBs (Fig. 2A–D), ceramides (Fig. 2E–F), and complex SPLs (Fig. 2G–I). Our results revealed distinct SPL profiles between the two mutants. In *orm1*Δ cells, levels of complex SPLs were elevated, indicating that excess LCBs are effectively funneled through the de novo pathway into complex SPLs, as expected (Fig. 2G–I). In contrast, *orm2*Δ cells exhibited the most pronounced changes at the level of LCBs and LCB-1-phosphates, with minimal changes in ceramide and complex SPL levels. The accumulation of LCBs in *orm2*Δ may be explained by the notion that Orm2 has a stronger inhibitory effect on SPT, and its deletion leads to elevated LCB production that exceeds the capacity of CerS to convert them into ceramides (11). However, the reduced level of ceramides and complex SPLs in *orm2*Δ compared to *orm1*Δ suggest an additional defect in the conversion of excess LCBs into ceramides (Fig. 2A–D).

To investigate this, we monitored the incorporation of deuterated D2-serine (L-serine (3,3)-D2), into the de novo SPL synthesis pathway. As shown in Figure 2J–N, deletion of *ORM1* did not lead to appreciable increases in LCBs or their phosphorylated derivatives (e.g., PHS-1P), but did result in elevated ceramide production. Conversely, *orm2*Δ cells showed limited ceramide accumulation and marked accumulation of D2-PHS and D2-PHS-1P. These findings suggest that while *orm1*Δ cells can efficiently convert LCBs into ceramides, *orm2*Δ cells display compromised ceramide synthase (CerS) activity, leading to impaired conversion of excess PHS. This is best illustrated by analyzing the product-to-substrate ratio of CerS, specifically the ratio of D2-C26-PHC to D2-PHS (Fig. 2O). This ratio was significantly lower in *orm2*Δ compared to WT or *orm1*Δ, the latter of which supports increased flux into ceramides without LCB accumulation. The observed CerS defect in *orm2*Δ is consistent with our previous report that LCB accumulation impairs CerS activity by reducing Ypk1-dependent Lac1 phosphorylation in *orm1/2*Δ (25). We did not detect a clear Lac1 phosphorylation defect in *orm2*Δ, likely because LCB accumulation is lower than in *orm1/2*Δ and the assay lacks sufficient sensitivity.

In summary, our results show that deletions of ORM1 and ORM2 have distinct effects on the SPL profile in yeast. While *orm1*Δ cells exhibit increased SPL production as expected, *orm2*Δ cells accumulate SPL precursors, LCBs, due to a defect in their conversion into ceramides.

### Orm2 controls Ypk1-dependent Orm1 phosphorylation through regulating LCB production

**Fig. 3:**
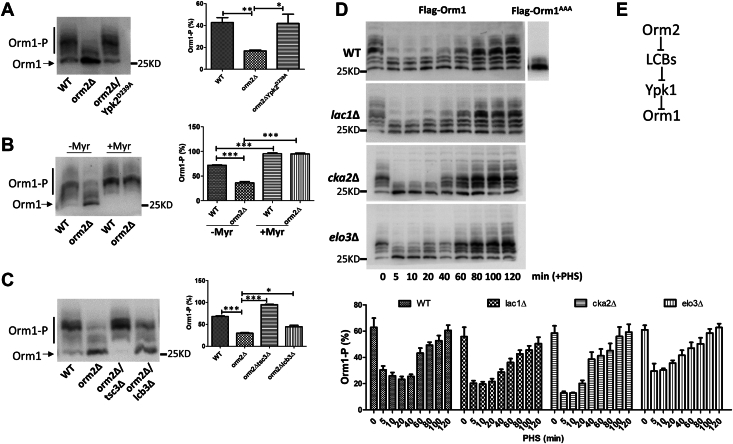
Orm2 regulates Orm1 phosphorylation through LCB-mediated Ypk1 activation. Whole-cell extracts from the indicated strains expressing Flag-tagged Orm1 or Flag-tagged Orm1^AAA^ were resolved via phos-tag SDS-PAGE followed by immunoblotting with an anti-Flag antibody. A: The Orm1 phosphorylation defect in *orm2Δ* cells was rescued by expression of the constitutively active Ypk2^D239A^. B: Treatment with myriocin (1μM) restored Orm1 phosphorylation in *orm2Δ* cells. C: Deletion of either *TSC3* or *LCB3* rescued and attenuated, respectively, the Orm1 phosphorylation defect in *orm2Δ*. D: Exogenously added PHS (10 μM) reduced Orm1 phosphorylation in all indicated strains. Yeast were collected at the indicated time points after PHS addition for analysis of Orm1 phosphorylation. Yeast expressing Flag-Orm1^AAA^ was included as a control to confirm Ypk1-phopshorylated Orm1 bands on Western blot. Western blots shown are representative of three independent biological replicates. Quantification of Orm1 phosphorylation (Orm1-P) is presented to the right for panels A–C and below for panel D. Orm1-P was quantified as the percentage intensity of the upper bands relative to the total Orm1 signal. Band intensities were measured using ImageJ. Statistical significance was determined using unpaired two-tailed t-tests in GraphPad Prism 5. ∗*P* < 0.05, ∗∗*P* < 0.01, ∗∗∗*P* < 0.001. E: Schematic model illustrating how Orm2 controls Orm1 phosphorylation through LCB-modulated Ypk1 activation.

It was observed previously that Orm1 phosphorylation changes in response to the status of Orm2: it decreases in *orm2*Δ cells and increases upon Orm2 overexpression or in the *orm2*^*AAA*^ mutant (4, 11). This was interpreted as a compensatory mechanism, whereby Orm1 adjusts its activity to offset the gain or loss of complex SPLs caused by alterations in Orm2 function. The Torc2-Ypk1 signaling pathway was proposed to mediate this response by sensing changes in complex SPL levels and modulating Orm1 phosphorylation accordingly (3, 9, 12, 13, 14). However, Orm2 does not alter its phosphorylation status in response to Orm1 gain- or loss-of-function (4, 11). This asymmetry is attributed to Orm2 being the predominant regulator of SPT activity, exerting a stronger influence on SPL production. However, our lipid analysis, showing accumulation of LCBs versus complex SPLs in *orm2*Δ versus *orm1*Δ, challenges this compensatory model and suggests that changes in LCB levels in *orm2*Δ regulate Orm1 phosphorylation status. To test this, we analyzed Orm1 phosphorylation status using a yeast strain expressing 3XFlag-tagged Orm1 as the sole copy of the *ORM1* gene (Fig. 3). Orm1 was resolved as multiple bands on the phos-tag gel, with the slower-migrating upper bands representing phosphorylated forms. These bands were absent in a non-phosphorylatable *orm1*^*AAA*^ mutant, in which Ypk1 phosphorylation sites (Ser51, Ser52, Ser53) were mutated to Alanine (Fig. 3D). Consistent with previous reports, Orm1 phosphorylation was markedly reduced in *orm2*Δ cells. This defect was rescued to wild-type levels by expressing Ypk2^D239A^, a constitutively active allele of the Ypk1 homologue Ypk2, demonstrating that impaired Ypk1 signaling indeed underlies the Orm1 phosphorylation defect in *orm2*Δ (Fig. 3A).

We next examined whether LCB accumulation in *orm2*Δ contributes to the Orm1 phosphorylation defect. Reducing LCB levels pharmacologically with the SPT inhibitor myriocin restored Orm1 phosphorylation to wild-type levels in *orm2*Δ (Fig. 3B). Similarly, genetically reducing LCB levels by deleting either TSC3 (encoding a small SPT subunit that positively regulates its activity) or LCB3 (encoding the LCB-1-P phosphatase) also restored Orm1 phosphorylation (Fig. 3C).

We further tested the effect of exogenously added phytosphingosine (PHS), the predominant LCB in yeast, on Orm1 phosphorylation. PHS treatment (10 μM) caused a rapid and transient decrease in Orm1 phosphorylation, with levels starting to drop within 5 min and recovering by ∼20 min after PHS treatment (Fig. 3D). This transient response suggests that the effect is mediated by PHS itself rather than downstream SPLs, as the timing of the loss of effect aligns with its conversion into ceramides. To confirm this, we assessed the effect of PHS on Orm1 phosphorylation in yeast strains with impaired ceramide synthesis (Fig. 3D). In *lac1*Δ (lacking a CerS catalytic subunit), *elo3*Δ (deficient in ceramide production due to impaired synthesis of very long-chain fatty acids), and *cka2*Δ (lacking a subunit of casein kinase 2 that is required for optimal CerS activity), PHS had a similar or even more pronounced effect on reducing Orm1 phosphorylation. We also exclude the possibility that the phosphorylation product of PHS, PHS-1-P, is the SPL intermediate controlling Orm1 phosphorylation in Figure 3C, by showing that in *lcb3*Δ—where deletion of the LCB-1-P phosphatase leads to increased PHS-1-P and decreased PHS—Orm1 phosphorylation is increased in *orm2Δ*. These results support the conclusion that PHS, rather than downstream ceramides or complex SPLs, regulates Ypk1-controlled Orm1 phosphorylation.

In summary, contrary to the prevailing belief that Ypk1-mediated changes in Orm1 phosphorylation in response to Orm2 status are driven by alterations in complex SPLs, our study reveals that PHS links Orm2 to Orm1 regulation by modulating Ypk1 signaling (Fig. 3E).

### Nitrogen induces increased SPL synthesis

Our findings challenge the compensatory model and suggest that Orm1 and Orm2 do not function merely to offset each other’s effects. Instead, the Orm2–LCB–Ypk1–Orm1 pathway may represent a distinct regulatory module with physiological significance. To test this, we first aimed to identify conditions under which SPL production is physiologically upregulated or downregulated, and then examined the specific roles of Orm1 and Orm2. Since our previous study implicated Orm proteins in endocytosis, we chose to investigate the yeast response to nitrogen availability, a condition known to regulate endocytosis of nutrient transporters. In yeast, as in higher eukaryotes, the serine/threonine kinase complex Target of Rapamycin Complex 1 (Torc1) plays a central role in mediating this response (30). Activated under nitrogen-rich conditions, Torc1 promotes anabolic processes to support cell growth and proliferation. While protein and lipid synthesis are known to be upregulated by Torc1, it remains unclear how SPL metabolism is regulated in response to nitrogen availability.

**Fig. 4:**
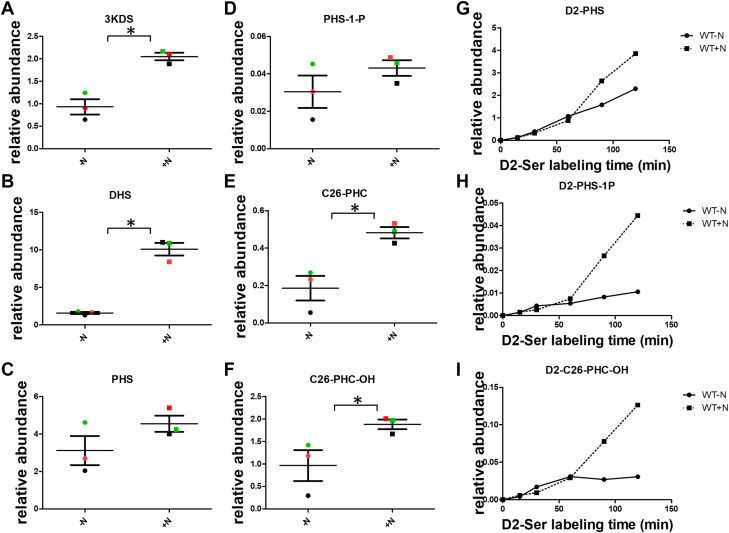
Nitrogen induces increased SPL production. A-F: SPL levels are elevated in yeast cultured in nitrogen-rich medium compared to nitrogen-poor medium. Whole-cell lipids were extracted from yeast grown for 2 h in the absence (-N) and presence (+) of 50 mM ammonium sulfate and analyzed by HPLC-ESI-MS/MS. The relative abundance of the indicated SPL species from three biological replicates is shown. ∗*P* < 0.05 by paired Student’s *t* test. G-I: Nitrogen enhances SPT activity. Incorporation of D2-Ser into LCB, D2-PHS (G); LCB-1P (D2-PHS-1P) (H); and ceramides (D2-C26-PHC-OH) (I) in yeast cultured under -N or +N conditions at the indicated time points. Shown are representative results from three independent experiments.

We first measured SPL level in yeast cultured in the presence (+N) and absence(-N) of ammonium sulfate. The -N is not nitrogen-free, as amino acids were added to meet the auxotrophic requirement of the strains; thus, we refer to -N as nitrogen-poor condition and +N as nitrogen-rich throughout this study. We quantified the steady state levels of LCBs (Fig. 4A–C), LCB-1-P (Fig. 4D) and ceramides (Fig. 4E–F) in yeast grown under both conditions. All SPL species measured showed a significant increase under the nitrogen-rich condition compared with nitrogen-poor condition. To determine whether this elevation in SPL levels under +N conditions results from increased SPT activity, we performed the D2-Ser labelling assay to assess in vivo SPT activity. Enhanced incorporation of D2-Ser into LCBs (D2-PHS; Fig. 4G), LCB-1-P (D2-PHS-1-P; Fig. 4H) and ceramides (D2-C26-PHC-OH; Fig. 4I) was observed in +N conditions. These findings indicate that SPT is activated in response to nitrogen abundance to promote SPL synthesis.

### Orm2 is required for nitrogen-induced SPL synthesis by regulating Ypk1-depenedent Orm1 phosphorylation

#### -Orm2 is required for nitrogen-induced Ypk1-dependent Orm1 phosphorylation

**Fig. 5:**
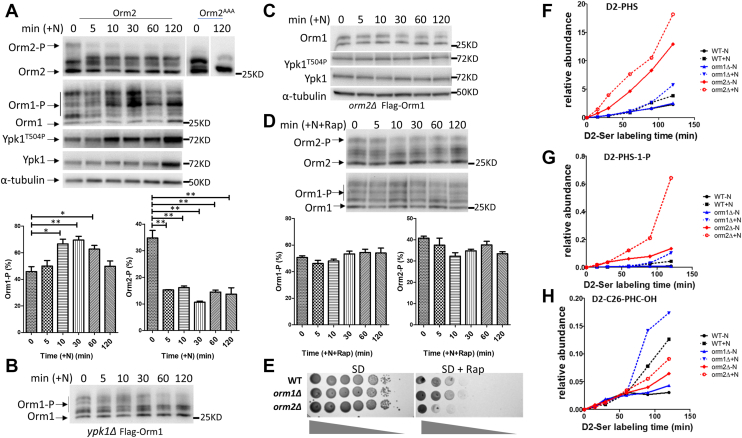
Orm2 is required for nitrogen-induced SPL synthesis by regulating Orm1-phosphorylation (inactivation) through Ypk1 signaling. A–C: Orm2 is required for nitrogen-induced Orm1 phosphorylation through Ypk1 signaling. Yeast was cultured in nitrogen-poor (-N) medium for 24 h before 50 mM ammonium sulfate (+N) was added for the indicated time points. Whole-cell protein extracts were analyzed by immunoblotting for the indicated proteins. A: Upon nitrogen supplementation, Orm2 is de-phosphorylated, Orm1 is phosphorylated, and Ypk1 is activated. Quantification of Orm1 and Orm2 phosphorylation (Orm1-P/Orm2-P) is presented below the blots. Orm1-P was quantified as the percentage intensity of the upper bands relative to the total Orm1 signal. Orm2-P was quantified as the percentage intensity of the top band relative to the total Orm2 signal. Band intensities were measured using ImageJ. Statistical significance was determined using unpaired two-tailed t-tests in GraphPad Prism 5. ∗*P* < 0.05, ∗∗*P* < 0.01. B: Orm1 phosphorylation decreases upon nitrogen supplementation in *ypk1Δ*. C: Ypk1 is not activated and Orm1 remains de-phosphorylated upon nitrogen supplementation in *orm2Δ*. D: Rapamycin treatment abolishes nitrogen-induced Orm2 dephosphorylation and Orm1 phosphorylation. Yeast cells were cultured in nitrogen-free (–N) medium for 24 h, followed by the addition of 50 mM ammonium sulfate for the indicated time points in the presence of 400 ng/ml rapamycin (Rap). Orm1/2 proteins from whole-cell lysates were detected by Western blot, and phosphorylation quantification is shown below. E: *orm1Δ* is resistant, while *orm2Δ* is sensitive to Rap. Serial dilutions of the indicated yeast strains were spotted on SD plate with or without 50 ng/ml Rap. Plates were imaged after 2 days of growth. F–H: Orm2, but not Orm1, is required for nitrogen-induced maximal SPT activation. Shown is the representative of three biological replicates. F, G: Accumulation of D2-PHS (F) and D2-PHS-1-P (G) is observed under both nitrogen-poor (−N) and nitrogen-replete (+N) conditions in *orm2Δ*, but not in *orm1Δ* or WT strains. H: Nitrogen-induced production of D2-ceramide (D2-C26-PHC-OH) is reduced in *orm2Δ* compared to WT and *orm1Δ*. Incorporation of D2-serine into the indicated sphingolipid intermediates was measured in WT, *orm1Δ*, and *orm2Δ* strains under −N and +N conditions at the indicated time points. Metabolites were analyzed by HPLC-ESI-MS/MS.

Next, we investigated the roles of Orm1 and Orm2 in nitrogen-induced SPL production. Cells were initially cultured under nitrogen-poor conditions, and upon nitrogen supplementation, we observed a rapid and sustained dephosphorylation of Orm2, beginning at 5 min and persisting throughout the time course examined (Fig. 5A). The top band of Orm2, which disappeared upon nitrogen addition, represents the Ypk1-phosphorylated form, as it was absent in the *orm2*^*AAA*^ mutant, where the Ypk1 phosphorylation sites (Ser46, Ser47, Ser48) are mutated to alanine. In contrast to Orm2, Orm1 phosphorylation increased in response to nitrogen, becoming evident at approximately 10 min after nitrogen supplementation (Fig. 5A). Around the same time, we detected activation of Ypk1, as indicated by increased phosphorylation at its activation site (T504) (Fig. 5A). At a later time point (120 min post-supplementation), we also observed elevated total Ypk1 protein levels. This is consistent with previous observations that Ypk1 upregulation accompanies its activation (25, 31). Together, these results reveal opposite phosphorylation responses of Orm1 and Orm2 to nitrogen repletion. This finding challenges the prevailing view that both Orm1 and Orm2 are phosphorylated in concert to promote SPL synthesis, and instead highlights a dynamic and opposing regulation of these two proteins.

We next investigated if the nitrogen-induced Orm1 phosphorylation is dependent on Ypk1. The results showed that no increase in Orm1 phosphorylation was observed in *ypk1Δ* (Fig. 5B). Instead, Orm1 phosphorylation gradually decreased, becoming apparent at the 60-min time point. Furthermore, in *orm2*Δ cells, only the lower bands of Orm1 were detected—similar to the *orm1*^*AAA*^ mutant—indicating that Orm1 remained in a dephosphorylated state (Fig. 5C). No increase in Orm1 phosphorylation was observed upon nitrogen supplementation, and Ypk1 activation was similarly absent in *orm2*Δ (Fig. 5C). These findings demonstrate Orm2 is required for nitrogen-induced Ypk1 activation and subsequent phosphorylation of Orm1. Together with previous observations showing that the non-phosphorylatable *orm2*^*AAA*^ mutant leads to a marked reduction in LCB levels (11), our results establish that the Orm2–LCB–Ypk1–Orm1 module is responsive to nitrogen availability. In this module, nitrogen addition triggers Orm2 dephosphorylation, lowering LCB levels and enabling Ypk1 activation to phosphorylate and inactivate Orm1.

#### -Rapamycin blocks nitrogen-induced Orm1/2 phosphorylation changes

Since Torc1 is a central regulator that coordinates cell growth with nutrient availability, we tested whether the Orm2 response to nitrogen is Torc1 dependent. In the presence of the Torc1 inhibitor, rapamycin, nitrogen-induced Orm2 dephosphorylation was blocked, and Orm1 phosphorylation was abolished (Fig. 5D), indicating that Torc1 mediates these phosphorylation events. Consistent with their opposite phosphorylation responses, *orm1*Δ and *orm2*Δ cells showed opposing sensitivities to rapamycin: *orm1*Δ cells were slightly more resistant, whereas *orm2*Δ cells were more sensitive (Fig. 5E). These results support distinct roles for Orm1 and Orm2 in promoting cell growth under nitrogen-rich conditions.

#### -Orm2 is required for nitrogen-induced SPL production

To test whether Orm2 and/or Orm1 are required for nitrogen-induced SPL synthesis, we examined the effects of deleting *ORM1* or *ORM2* on SPL production following nitrogen supplementation. In WT cells, D2-serine labeling assays revealed increased incorporation of D2-Ser into long-chain bases (D2-PHS; Fig. 5F), LCB-1-phosphates (D2-PHS-1-P; Fig. 5G), and ceramides (D2-C26-PHC-OH; Fig. 5H), with the most pronounced increase observed in ceramide levels. In *orm2*Δ cells, although basal levels of LCBs, LCB-1-Ps, and ceramides were higher under nitrogen-poor conditions, the increase in ceramide production upon nitrogen supplementation was significantly less than in WT, resulting in the lowest ceramide levels under nitrogen-rich conditions. By contrast, the *orm1*Δ mutant showed a similar response to WT, exhibiting a marked increase in ceramide production following nitrogen addition. These results highlight distinct roles for Orm1 and Orm2 in nitrogen-induced SPL synthesis. While Orm1 inactivation facilitates SPL production, Orm2 is essential for activating this process. Notably, the comparable increases in LCB and LCB-1-P levels across WT, *orm1Δ*, and *orm2Δ* strains, along with the observation that *ORM1* deletion alone does not restore SPL synthesis under nitrogen-poor conditions to the levels seen under nitrogen-rich conditions, suggest that additional, Orm-independent mechanisms may contribute to nitrogen-induced sphingolipid synthesis.

### Orm1 and Orm2 play distinct roles in nitrogen-induced endocytosis of Gap1

**Fig. 6:**
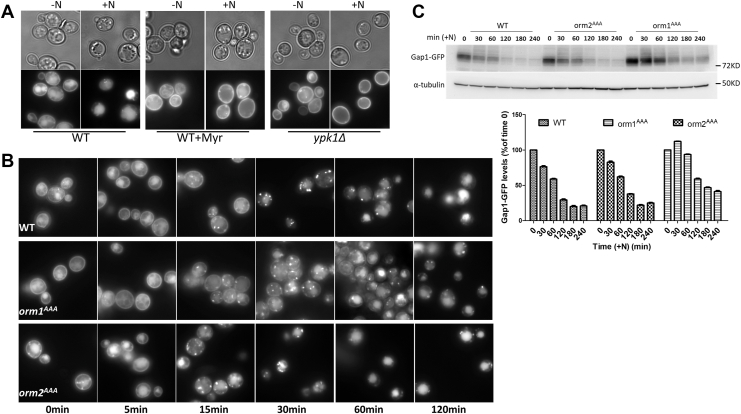
Orm1 and Orm2 play distinct role in nitrogen-induced endocytosis of Gap1. A: Nitrogen-induced endocytosis of Gap1 is blocked when SPL synthesis is impaired. Gap1-GFP localization was examined in the indicated yeast strains cultured in the presence or absence of 50 mM ammonium sulfate and 1 μM myriocin for 2 h. Cells were imaged using differential interference contrast (DIC) (top) and fluorescence microscopy (480 nm excitation/510 nm emission) (bottom). B-C: Nitrogen-induced endocytosis of Gap1 is impaired in the *orm1*^*AAA*^ mutant but not in the *orm2*^*AAA*^ mutant. Yeast strains were cultured in nitrogen-poor medium for 24 h prior to ammonium sulfate supplementation. Gap1-GFP localization was monitored by fluorescence microscopy at the indicated time points (B). Gap1-GFP levels were assessed by Western blotting after SDS-PAGE of whole-cell protein extracts (C). Quantification of Western blotting is shown below, with values expressed as percentage relative to the signal detected at time 0. Gap1-GFP signals were normalized to the loading control (α-tubulin).

SPLs have been implicated in regulating endocytosis in both yeast and mammalian systems, where impaired SPL synthesis leads to disrupted endocytosis of yeast pheromone α-factor, the Serotonin_1A_ receptor and the Wnt ligand Wingless (32, 33, 34, 35, 36). In this study, we focused on the general amino acid permease Gap1, whose endocytosis is acutely regulated by nitrogen availability. Under nitrogen-free conditions, Gap1 localizes to the plasma membrane (PM), purportedly to scavenge any residual nitrogen from the environment. When nitrogen becomes abundant, Gap1 is rapidly internalized and delivered to the vacuole (the yeast equivalent of the lysosome) (37, 38). Here, we monitored Gap1-GFP localization to assess the role of SPLs in endocytosis. In nitrogen-poor medium, Gap1-GFP was detected at the PM, with some accumulation in the vacuole—due to the medium used here not being completely nitrogen-free. Upon nitrogen supplementation, Gap1-GFP was fully redistributed to the vacuole, and the PM signal was lost (Fig. 6A). This nitrogen-induced translocation was abolished by myriocin treatment, resulting in Gap1-GFP being retained at the PM. A similar defect in Gap1-GFP endocytosis was observed in *ypk1*Δ mutants, which have severely impaired SPL synthesis due to reduced SPT and CerS activity (9, 12, 14). Notably, under nitrogen-poor conditions, both *ypk1Δ* and myriocin-treated WT cells showed markedly reduced accumulation of Gap1-GFP in the vacuole. These observations indicate that SPLs are necessary for endocytosis of Gap1.

In Figure 5, the results show that Orm2 is required for nitrogen-induced SPL production by promoting Orm1 phosphorylation. Here, we tested whether Gap1 endocytosis is impaired in the non-phosphorylatable *orm1*^*AAA*^ mutant. Yeast cells were first cultured in nitrogen-poor medium, and Gap1-GFP signal was visualized after ammonium sulfate was added. In WT cells, the PM fraction of Gap1-GFP gradually disappeared over 120 min (Fig. 6B). Punctate structures, likely representing endosomes, appeared around 15 min and persisted until 60 min. By 120 min, GFP signals were predominantly in the vacuole. In the *orm1*^*AAA*^ mutant, this process was delayed: PM-associated Gap1-GFP remained visible up to 60 min, and punctate structures were still detectable at 120 min—unlike in WT. In contrast, the *orm2*^*AAA*^ mutant showed Gap1-GFP endocytosis kinetics similar to WT. These observations were corroborated by Western blot analysis, which revealed a slower degradation of full-length Gap1-GFP over 240 min in *orm1*^*AAA*^ but not *orm2*^*AAA*^ mutant (Fig. 6C). These findings indicate that Orm1 and Orm2 play distinct roles in nitrogen-induced endocytosis of Gap1, consistent with their differing contributions to sphingolipid production.

## Discussion

The Orm family proteins are evolutionarily conserved negative regulators of SPT. In humans, these proteins, particularly *ORMDL3*, have been linked to multiple diseases and are thought to control inflammatory responses by maintaining calcium homeostasis and regulating the endoplasmic reticulum (ER) stress response (39, 40, 41, 42, 43, 44). However, it remains unclear whether these effects are mediated through the regulation of SPL synthesis. This uncertainty stems largely from the limited understanding of how individual Orm protein control SPL production, beyond the assumption that they have overlapping functions.

**Fig. 7:**
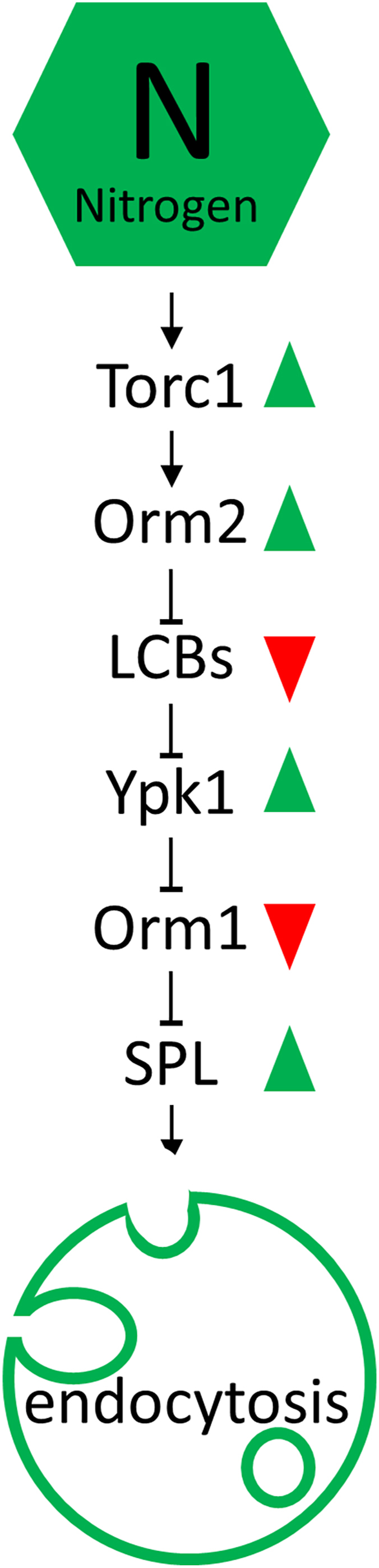
Schematic model of how the Orm2–LCB–Ypk1–Orm1 regulatory module mediates nitrogen-induced sphingolipid synthesis and endocytosis. Pointed and flat arrows between nodes indicate activation and inhibition, respectively. Green and red arrowheads next to each node denote increased and decreased activity or levels. See text for details.

In this study, we reveal that yeast Orm2 modulates production of complex SPLs indirectly by controlling LCB-modulated Ypk1 activation (Fig. 7). Furthermore, we demonstrate that this newly elucidated Orm2-LCB-Ypk1-Orm1 regulatory module links Torc1-controlled nitrogen response to SPL production and endocytosis.

### Orm2-LCB-Ypk1-Orm1 represents a regulatory module

Alterations in Orm1 phosphorylation in response to changes in Orm2 function have been interpreted as a compensatory mechanism, in which Orm1 modulates its activity to balance the increase or decrease in complex sphingolipids resulting from Orm2 dysregulation. Here, we propose that this actually represents a regulatory module where Orm2 controls Orm1 phosphorylation through LCB-mediated Ypk1 activation.

The prevailing assumption has been that both Orm proteins regulate the production of complex SPLs, with Orm2 playing the dominant role. This view is based on observations that LCB levels are altered in *orm2* mutant but not in *orm1* mutant and on the presumption that LCB levels directly reflect complex SPL levels. However, the current data show that LCB levels do not reliably predict complex SPL levels. Specifically, complex SPLs increase in *orm1*Δ mutants without corresponding changes in LCBs. On the other hand, LCBs accumulate in *orm2*Δ cells without affecting complex SPL levels. Therefore, the two Orms exhibit distinct functional roles in sphingolipid biosynthesis. Together with previous findings demonstrating that LCB accumulation impairs Ypk1 signaling in *orm1/2*Δ mutants, the current results indicate that Orm2 influences Orm1 phosphorylation through LCB-mediated regulation of Ypk1 activity. This defines an Orm2-LCB-Ypk1-Orm1 regulatory module.

In this study, we further demonstrate that the Orm2-LCB-Ypk1-Orm1 module is responsive to nitrogen availability to regulate SPL production. Contrary to its previously assumed inhibitory role, Orm2 promotes SPL production upon nitrogen supplementation by facilitating Orm1 phosphorylation via the Ypk1 kinase. Specifically, nitrogen addition leads to Orm2 dephosphorylation and activation, resulting in decreased levels of LCBs. This reduction in LCBs relieves the inhibition on Ypk1, allowing Ypk1 to phosphorylate and inactivate Orm1, thereby promoting SPL biosynthesis (Fig. 7).

The connection between Torc1, yeast Orm proteins, and SPLs has been proposed previously without definitive conclusions. One report claimed that rapamycin treatment induces phosphorylation of Orm1 but not Orm2 (45). Another reported that both Orm proteins are phosphorylated at N-terminal serine/threonine residues—distinct from the Ypk1 target sites—upon rapamycin treatment, and that this phosphorylation promotes sphingolipid synthesis independently of SPT (46). In this study, we conclude that nitrogen promotes sphingolipid synthesis in a Torc1-dependent manner, as evidenced by increased SPL production and changes in Orm protein phosphorylation upon nitrogen supplementation. We focus on the well-characterized Ypk1 phosphorylation sites and show that Orm2 phosphorylation is rapidly reduced upon nitrogen supplementation. This dephosphorylation is abolished by rapamycin treatment, indicating a Torc1-dependent process. We further demonstrate that Orm2 dephosphorylation is necessary for subsequent Ypk1 activation, Orm1 phosphorylation, and sphingolipid production. The observed activation of Ypk1 following nitrogen addition suggests that the reduced Orm2 phosphorylation likely results from phosphatase activation. Future studies will explore the mechanisms by which Torc1 regulates Orm2 dephosphorylation.

These findings suggest that Orm2 primarily functions as a modulator of Ypk1 activity by regulating the production of LCBs.Ypk1 not only phosphorylates Orm proteins and ceramide synthase to regulate SPL synthesis, but it also targets other substrates involved in diverse cellular functions (47, 48, 49, 50, 51, 52, 53, 54, 55, 56, 57). Given that Orm2 is regulated at both translational and post-translational levels —upregulated in response to ER stress (4) and dephosphorylated following nitrogen supplementation (this study)—our findings suggest that Orm2 primarily functions as a regulatory protein that integrates environmental and cellular stress cues to modulate Ypk1 signaling. Future studies will investigate whether, and how, Orm2 contributes to other Ypk1-regulated cellular processes.

In summary, our study supports a model in which Orm2, LCBs, Ypk1, and Orm1 act together as a regulatory module, rather than as components of a simple compensatory feedback loop.

### SPLs and Endocytosis of Gap1

Endocytosis of the general amino acid permease Gap1 in response to nitrogen supplementation is known to be regulated by Torc1-dependent ubiquitination (38, 58). In this study, we uncover an additional layer of regulation by demonstrating that SPLs are required for Gap1 endocytosis. Inhibiting SPL biosynthesis—either pharmacologically with myriocin or genetically by deleting YPK1—markedly impairs nitrogen-induced endocytosis of Gap1. This is consistent with previous reports showing that SPLs are critical for the endocytosis of PM proteins, including transporters, receptors, and ion channels, across various systems (32, 33, 34, 35, 59). Compared to the complete block of Gap1 endocytosis observed in *ypk1Δ* and myriocin-treated cells, the *orm1*^*AAA*^ mutant, which prevents Ypk1-dependent Orm1 phosphorylation, showed only a delay in Gap1 internalization. This could be explained by *ypk1*Δ and myriocin having a more profound impact by suppressing basal SPL levels, whereas *orm1*^*AAA*^ primarily impairs the nitrogen-induced upregulation of SPL synthesis. Although Orm2 is required for Orm1 phosphorylation and full activation of SPL synthesis upon nitrogen repletion, we observed a modest increase in SPL levels even in the *orm2*Δ mutant after nitrogen addition. This indicates that Orm proteins are not the sole regulators of nitrogen-induced SPL synthesis. Nitrogen may also promote SPL production by increasing the synthesis or uptake of SPT substrates serine and palmitoyl-CoA.

In summary, this study reveals that Orm1 and Orm2 play distinct roles in SPL regulation. While Orm1 acts as a negative regulator of SPL synthesis by directly inhibiting SPT, Orm2 promotes SPL production indirectly through an LCB–Ypk1–Orm1 signaling module. This regulatory circuit is activated in response to nitrogen availability and is required for both SPL synthesis and the endocytosis of the general amino acid permease Gap1. Our findings suggest that, beyond maintaining SPL homeostasis, certain Orm proteins—such as Orm2—may function as upstream regulators of Ypk1 signaling in response to environmental cues.

## Data availability

All data are contained within the manuscript.

## Conflict of Interest

The authors declare that they do not have any conflicts of interest with the content of this article.

## References

[1] Hannun Y.A., Obeid L.M. (2018). Sphingolipids and their metabolism in physiology and disease. Nat. Rev. Mol. Cell Biol..

[2] Ren J., Hannun Y.A., Geiger O. (2017). Biogenesis of Fatty Acids, Lipids and Membranes.

[3] Breslow D.K., Collins S.R., Bodenmiller B., Aebersold R., Simons K., Shevchenko A. (2010). Orm family proteins mediate sphingolipid homeostasis. Nature.

[4] Han S., Lone M.A., Schneiter R., Chang A. (2010). Orm1 and Orm2 are conserved endoplasmic reticulum membrane proteins regulating lipid homeostasis and protein quality control. Proc. Natl. Acad. Sci. U. S. A..

[5] Hjelmqvist L., Tuson M., Marfany G., Herrero E., Balcells S., Gonzalez-Duarte R. (2002). ORMDL proteins are a conserved new family of endoplasmic reticulum membrane proteins. Genome Biol..

[6] Li S., Xie T., Liu P., Wang L., Gong X. (2021). Structural insights into the assembly and substrate selectivity of human SPT-ORMDL3 complex. Nat. Struct. Mol. Biol..

[7] Schafer J.H., Korner C., Esch B.M., Limar S., Parey K., Walter S. (2023). Structure of the ceramide-bound SPOTS complex. Nat. Commun..

[8] Han G., Gupta S.D., Gable K., Bacikova D., Sengupta N., Somashekarappa N. (2019). The ORMs interact with transmembrane domain 1 of Lcb1 and regulate serine palmitoyltransferase oligomerization, activity and localization. Biochim. Biophys. Acta Mol. Cell Biol. Lipids.

[9] Roelants F.M., Breslow D.K., Muir A., Weissman J.S., Thorner J. (2011). Protein kinase Ypk1 phosphorylates regulatory proteins Orm1 and Orm2 to control sphingolipid homeostasis in Saccharomyces cerevisiae. Proc. Natl. Acad. Sci. U. S. A..

[10] Schmidt O., Weyer Y., Baumann V., Widerin M.A., Eising S., Angelova M. (2019). Endosome and Golgi-associated degradation (EGAD) of membrane proteins regulates sphingolipid metabolism. EMBO J..

[11] Korner C., Schafer J.H., Esch B.M., Parey K., Walter S., Teis D. (2024). The structure of the Orm2-containing serine palmitoyltransferase complex reveals distinct inhibitory potentials of yeast Orm proteins. Cell Rep..

[12] Aronova S., Wedaman K., Aronov P.A., Fontes K., Ramos K., Hammock B.D. (2008). Regulation of ceramide biosynthesis by TOR complex 2. Cell Metab..

[13] Berchtold D., Piccolis M., Chiaruttini N., Riezman I., Riezman H., Roux A. (2012). Plasma membrane stress induces relocalization of Slm proteins and activation of TORC2 to promote sphingolipid synthesis. Nat. Cel. Biol..

[14] Muir A., Ramachandran S., Roelants F.M., Timmons G., Thorner J. (2014). TORC2-dependent protein kinase Ypk1 phosphorylates ceramide synthase to stimulate synthesis of complex sphingolipids. eLife.

[15] Green C.D., Weigel C., Oyeniran C., James B.N., Davis D., Mahawar U. (2021). CRISPR/Cas9 deletion of ORMDLs reveals complexity in sphingolipid metabolism. J. Lipid Res..

[16] Zhakupova A., Debeuf N., Krols M., Toussaint W., Vanhoutte L., Alecu I. (2016). ORMDL3 expression levels have no influence on the activity of serine palmitoyltransferase. FASEB J..

[17] Mathivanan A., Nachiappan V. (2023). Deletion of ORM2 causes oleic acid-induced growth defects in Saccharomyces cerevisiae. Appl. Biochem. Biotechnol..

[18] Lee Y.J., Huang X., Kropat J., Henras A., Merchant S.S., Dickson R.C. (2012). Sphingolipid signaling mediates iron toxicity. Cell Metab..

[19] Moffatt M.F., Kabesch M., Liang L., Dixon A.L., Strachan D., Heath S. (2007). Genetic variants regulating ORMDL3 expression contribute to the risk of childhood asthma. Nature.

[20] Stein M.M., Thompson E.E., Schoettler N., Helling B.A., Magnaye K.M., Stanhope C. (2018). A decade of research on the 17q12-21 asthma locus: piecing together the puzzle. J. Allergy Clin. Immunol..

[21] McGovern D.P., Gardet A., Torkvist L., Goyette P., Essers J., Taylor K.D. (2010). Genome-wide association identifies multiple ulcerative colitis susceptibility loci. Nat. Genet..

[22] Hoefkens E., Nys K., John J.M., Van Steen K., Arijs I., Van der Goten J. (2013). Genetic association and functional role of Crohn disease risk alleles involved in microbial sensing, autophagy, and endoplasmic reticulum (ER) stress. Autophagy.

[23] Yang W., Sheng F., Sun B., Fischbach S., Xiao X. (2019). The role of ORMDL3/ATF6 in compensated beta cell proliferation during early diabetes. Aging (Albany NY).

[24] Lee H., Fenske R.J., Akcan T., Domask E., Davis D.B., Kimple M.E. (2020). Differential expression of ormdl genes in the islets of mice and humans with obesity. iScience.

[25] Ren J., Rieger R., Pereira de Sa N., Kelapire D., Del Poeta M., Hannun Y.A. (2024). Orm proteins control ceramide synthesis and endocytosis via LCB-mediated Ypk1 regulation. J. Lipid Res..

[26] Longtine M.S., McKenzie A., Demarini D.J., Shah N.G., Wach A., Brachat A. (1998). Additional modules for versatile and economical PCR-based gene deletion and modification in Saccharomyces cerevisiae. Yeast.

[27] Ren J., Saied E.M., Zhong A., Snider J., Ruiz C., Arenz C. (2018). Tsc3 regulates SPT amino acid choice in Saccharomyces cerevisiae by promoting alanine in sphingolipid pathway. J. Lipid Res..

[28] Ren J., Snider J., Airola M.V., Zhong A., Rana N.A., Obeid L.M. (2018). Quantification of 3-ketodihydrosphingosine using HPLC-ESI-MS/MS to study SPT activity in yeast Saccharomyces cerevisiae. J. Lipid Res..

[29] Ishino Y., Komatsu N., Sakata K.T., Yoshikawa D., Tani M., Maeda T. (2022). Regulation of sphingolipid biosynthesis in the endoplasmic reticulum via signals from the plasma membrane in budding yeast. FEBS J..

[30] Battaglioni S., Benjamin D., Walchli M., Maier T., Hall M.N. (2022). mTOR substrate phosphorylation in growth control. Cell.

[31] Schmidt O., Weyer Y., Sprenger S., Widerin M.A., Eising S., Baumann V. (2020). TOR complex 2 (TORC2) signaling and the ESCRT machinery cooperate in the protection of plasma membrane integrity in yeast. J. Biol. Chem..

[32] Cheng Z.J., Singh R.D., Sharma D.K., Holicky E.L., Hanada K., Marks D.L. (2006). Distinct mechanisms of clathrin-independent endocytosis have unique sphingolipid requirements. Mol. Biol. Cel..

[33] Kumar A., Sarkar P., Chattopadhyay A. (2023). Metabolic depletion of sphingolipids inhibits agonist-induced endocytosis of the serotonin(1A) receptor. Traffic.

[34] Pepperl J., Reim G., Luthi U., Kaech A., Hausmann G., Basler K. (2013). Sphingolipid depletion impairs endocytic traffic and inhibits Wingless signaling. Mech. Dev..

[35] Zanolari B., Friant S., Funato K., Sutterlin C., Stevenson B.J., Riezman H. (2000). Sphingoid base synthesis requirement for endocytosis in Saccharomyces cerevisiae. EMBO J..

[36] Chung N., Jenkins G., Hannun Y.A., Heitman J., Obeid L.M. (2000). Sphingolipids signal heat stress-induced ubiquitin-dependent proteolysis. J. Biol. Chem..

[37] Lauwers E., Erpapazoglou Z., Haguenauer-Tsapis R., Andre B. (2010). The ubiquitin code of yeast permease trafficking. Trends Cell Biol..

[38] Merhi A., Andre B. (2012). Internal amino acids promote Gap1 permease ubiquitylation via TORC1/Npr1/14-3-3-dependent control of the Bul arrestin-like adaptors. Mol. Cell. Biol..

[39] Cantero-Recasens G., Fandos C., Rubio-Moscardo F., Valverde M.A., Vicente R. (2010). The asthma-associated ORMDL3 gene product regulates endoplasmic reticulum-mediated calcium signaling and cellular stress. Hum. Mol. Genet..

[40] Carreras-Sureda A., Cantero-Recasens G., Rubio-Moscardo F., Kiefer K., Peinelt C., Niemeyer B.A. (2013). ORMDL3 modulates store-operated calcium entry and lymphocyte activation. Hum. Mol. Genet..

[41] Dang J., Bian X., Ma X., Li J., Long F., Shan S. (2017). ORMDL3 facilitates the survival of splenic B cells via an ATF6alpha-Endoplasmic reticulum Stress-Beclin1 autophagy regulatory pathway. J. Immunol..

[42] Hsu K.J., Turvey S.E. (2013). Functional analysis of the impact of ORMDL3 expression on inflammation and activation of the unfolded protein response in human airway epithelial cells. Allergy Asthma. Clin. Immunol..

[43] Schmiedel B.J., Seumois G., Samaniego-Castruita D., Cayford J., Schulten V., Chavez L. (2016). 17q21 asthma-risk variants switch CTCF binding and regulate IL-2 production by T cells. Nat. Commun..

[44] Zhang Y., Willis-Owen S.A.G., Spiegel S., Lloyd C.M., Moffatt M.F., Cookson W. (2019). The ORMDL3 asthma gene regulates ICAM1 and has multiple effects on cellular inflammation. Am. J. Respir. Crit. Care Med..

[45] Liu M., Huang C., Polu S.R., Schneiter R., Chang A. (2012). Regulation of sphingolipid synthesis through Orm1 and Orm2 in yeast. J. Cell Sci..

[46] Shimobayashi M., Oppliger W., Moes S., Jeno P., Hall M.N. (2013). TORC1-regulated protein kinase Npr1 phosphorylates Orm to stimulate complex sphingolipid synthesis. Mol. Biol. Cel..

[47] Roelants F.M., Baltz A.G., Trott A.E., Fereres S., Thorner J. (2010). A protein kinase network regulates the function of aminophospholipid flippases. Proc. Natl. Acad. Sci. U. S. A..

[48] Lee Y.J., Jeschke G.R., Roelants F.M., Thorner J., Turk B.E. (2012). Reciprocal phosphorylation of yeast glycerol-3-phosphate dehydrogenases in adaptation to distinct types of stress. Mol. Cell. Biol..

[49] Alvaro C.G., Aindow A., Thorner J. (2016). Differential phosphorylation provides a switch to control how alpha-Arrestin Rod1 down-regulates mating pheromone response in Saccharomyces cerevisiae. Genetics.

[50] Galez H.A., Roelants F.M., Palm S.M., Reynaud K.K., Ingolia N.T., Thorner J. (2021). Phosphorylation of mRNA-Binding proteins Puf1 and Puf2 by TORC2-Activated protein kinase Ypk1 alleviates their repressive effects. Membranes (Basel).

[51] deHart A.K., Schnell J.D., Allen D.A., Hicke L. (2002). The conserved Pkh-Ypk kinase cascade is required for endocytosis in yeast. J. Cel. Biol..

[52] Muir A., Roelants F.M., Timmons G., Leskoske K.L., Thorner J. (2015). Down-regulation of TORC2-Ypk1 signaling promotes MAPK-independent survival under hyperosmotic stress. eLife.

[53] Mulet J.M., Martin D.E., Loewith R., Hall M.N. (2006). Mutual antagonism of target of rapamycin and calcineurin signaling. J. Biol. Chem..

[54] Niles B.J., Joslin A.C., Fresques T., Powers T. (2014). TOR complex 2-Ypk1 signaling maintains sphingolipid homeostasis by sensing and regulating ROS accumulation. Cell Rep..

[55] Schmidt A., Kunz J., Hall M.N. (1996). TOR2 is required for organization of the actin cytoskeleton in yeast. Proc. Natl. Acad. Sci. U. S. A..

[56] Schonbrun M., Kolesnikov M., Kupiec M., Weisman R. (2013). TORC2 is required to maintain genome stability during S phase in fission yeast. J. Biol. Chem..

[57] Vlahakis A., Graef M., Nunnari J., Powers T. (2014). TOR complex 2-Ypk1 signaling is an essential positive regulator of the general amino acid control response and autophagy. Proc. Natl. Acad. Sci. U. S. A..

[58] MacGurn J.A., Hsu P.C., Smolka M.B., Emr S.D. (2011). TORC1 regulates endocytosis via Npr1-mediated phosphoinhibition of a ubiquitin ligase adaptor. Cell.

[59] Rispal D., Eltschinger S., Stahl M., Vaga S., Bodenmiller B., Abraham Y., Filipuzzi I., Movva N.R., Aebersold R., Helliwell S.B., Loewith R. (2015). Target of Rapamycin complex 2 regulates actin polarization and endocytosis via multiple pathways. J. Biol. Chem..

